# Society of Alumni of the Atlanta Medical College

**Published:** 1857-08

**Authors:** 


### SOCIETY OF ALUMNI OF THE ATLANTA MEDICAL COLLEGE.

☞ We hope it will be recollected by the graduates of this school that at the close of the last session a "*Society of Alumni*" was organized and a speaker appointed to address them at the close of the present Session upon the occasion of their Anniversary Meeting, which will take place on the *night* of the 3d of September, that being the *day* upon which the degrees will be conferred, and the Valedictory address delivered to the Graduating Class, in the Atlanta Medical College. The anniversary address to the Society of Alumni will be delivered by N. F. Powers, M. D. of Thomson, Ga., who will doubtless make it an occasion of interest to all who attend, and we cannot too strongly urge upon the *alumni* of the Institution the importance of meeting their brethren upon that occasion. We hope to see a large number of those who have thus become identified with the welfare of this college present at the commencement, not only for the purpose of reneiwng the friendship of former days, but that we may be able to show them that we have not been idle or resting upon the achievments of our first years, but that our motto has been *onward* ; until now, we do not shrink from a comparison of our facilities for medical teaching with those of any institution in the land.

